# The effects of kinesiology taping on experimentally-induced thermal and mechanical pain in otherwise pain-free healthy humans: A randomised controlled repeated-measures laboratory study

**DOI:** 10.1371/journal.pone.0226109

**Published:** 2019-12-10

**Authors:** Gourav Banerjee, Michelle Briggs, Mark I. Johnson

**Affiliations:** 1 Centre for Pain Research, School of Clinical and Applied Sciences, Leeds Beckett University, Leeds, England, United Kingdom; 2 Division of Nursing, Midwifery and Social Work, School of Health Sciences, Faculty of Biology, Medicine and Health, University of Manchester, Manchester Academic Health Science Centre, Manchester, England, United Kingdom; University of Ottawa, CANADA

## Abstract

**Background:**

Kinesiology taping (KT) is used to manage musculoskeletal-related pain. There is a paucity of physiological studies evaluating the effect of KT on stimulus-evoked experimental pain.

**Objective:**

To investigate the effect of KT (applied to lumbar region) on cutaneous somatosensation to noxious and innocuous stimuli in humans with a non-sensitised normally functioning nociceptive system using quantitative sensory testing (QST).

**Methods:**

Fifty-four participants were randomised to one of three interventions: (i) KT (ii) standard ‘rigid’ taping (ST) (iii) sham taping (ShT). QST measurements were taken at lumbar sites pre-intervention (T1), during-intervention (T2) and during-intervention (T3) in the following sequence: warm-detection-threshold (WDT), heat-pain-threshold (HTPh), heat-pain-tolerance (HPTo), mechanical-detection-threshold (MDT), mechanical-pain-threshold (MPT) and pressure-pain-threshold (PPT).

**Results:**

Mixed ANOVA revealed statistically significant interaction between Intervention and Time on MDT (*p* < .0005) and MPT (*p* < .0005) but not on WDT (*p* = .09), HPTh (*p* = .09), HPTo (*p* = .51) and PPT (*p* = .52) datasets. There was no significant simple main effect of Intervention on MDT at T2 (*p* = .68) and T3 (*p* = .24), and MPT at T2 (*p* = .79) and T3 (*p* = .54); post-hoc tests found KT and ST groups had higher (but non-significant) MDT and MPT than the ShT group. There was a significant simple main effect of Time on MDT and MPT for KT (*p* < .0005) and ST (*p* < .0005) groups; post-hoc tests found significant increases in MDT and MPT at T3 and T2 compared with T1 in both KT and ST groups. There was no significant simple main effect of Time on MDT (*p* = .13) nor MPT (*p* = .08) for the ShT group.

**Conclusion:**

Taping, irrespective of the elasticity, may modulate cutaneous mechanosensation. KT, ST and ShT seemed to have similar influence on cutaneous thermal and deep pressure nociception.

## Introduction

**Fig 1:**
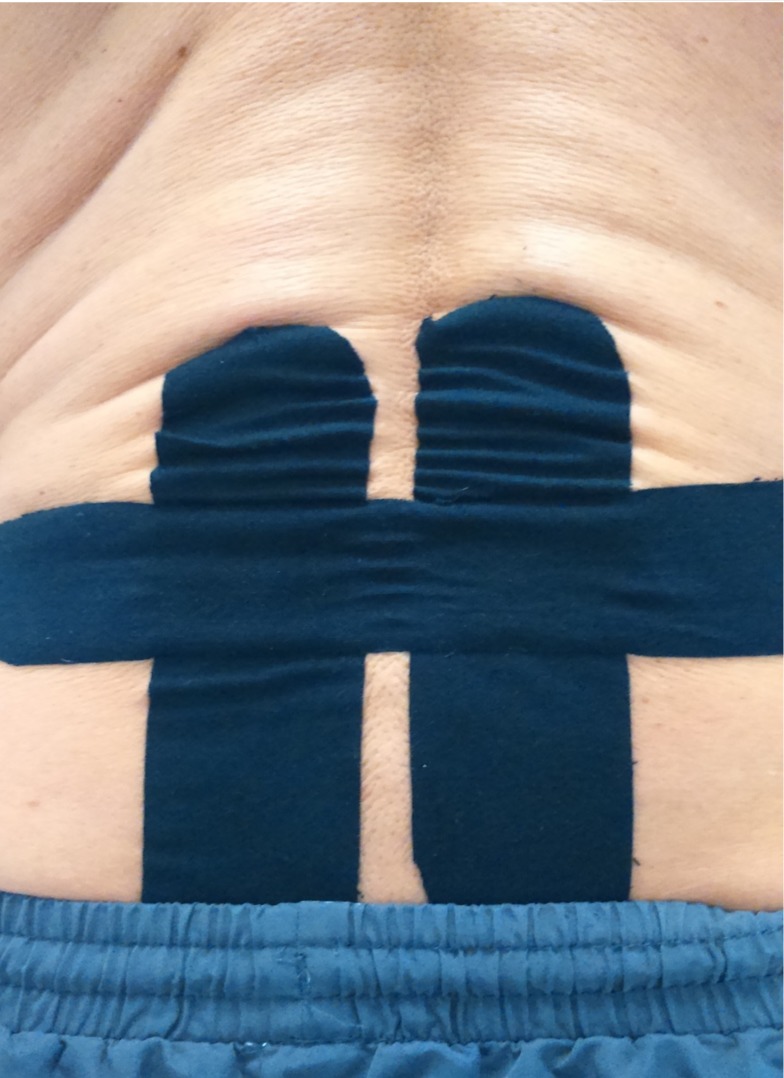
Figure showing visible convolutions of skin upon kinesiology taping.

Kinesiology taping is used in musculoskeletal practice and sports settings by healthcare professionals for the management of pain and the prevention and rehabilitation of injuries. There is tentative evidence from recent systematic reviews with meta-analysis that kinesiology taping may reduce myofascial pain and pain in the lower back, shoulder and knee regions and improve functional outcomes in the short-term [1–5]. Kinesiology taping involves the application of thin, elastic, cotton-based water-resistant adhesive kinesiology tape to the skin. The properties of kinesiology tape allow the tape to be stretched longitudinally up to 60% or more of its resting length and worn continuously for 3–5 days to support soft tissues and joints whilst not restricting movements. Kinesiology taping produces mechanical deformation of tissues underneath the tape in humans [6,7] and may generate visible convolutions of skin when applied to certain areas of the body such as lower back (Fig 1). It is claimed that the elastic nature of kinesiology tape generates stretching and recoiling of the skin and superficial tissues during movement, resulting in mechanical deformation and stimulation of low‑threshold mechanoreceptors in the skin, fascia, Golgi tendon organs and (skeletal) muscle spindles [1,2,8–10]. It is claimed that activation of low threshold mechanoreceptor peripheral afferents during kinesiology taping leads to inhibition of ongoing nociceptive transmission from centrally transmitting nociceptive cells, in line with the gate control theory of pain [11]. There is a paucity of physiological investigations into the effects of kinesiology taping on pain and somatosensory function.

Studies exposing healthy participants to experimentally-induced pain are used to study mechanisms and outcomes of treatments because they enable greater control of factors that confound clinical studies [12,13]. There are conflicting findings of studies that have evaluated the effect of kinesiology taping on transient nociceptive-stimuli interacting with a normally functioning nociceptive system in the absence or presence of sensitisation. For example, it has been found that kinesiology taping reduced experimentally-induced delayed onset muscle soreness (DOMS) in some studies [14–17] but not in others [18–20]. Psychophysical techniques such as quantitative sensory testing (QST) can be used to evaluate the effect of kinesiology taping on perceptual experiences associated with primary afferent fibre activity. Clinically, QST is employed to quantify somatosensory function in individuals with suspected pathology involving nerve fibre integrity using the individual’s subjective response to calibrated and controlled stimuli applied to the skin, mucosa or muscle tissue. QST can also be used to evaluate the effect of treatments on somatosensation, including pain, on pain-free healthy individuals. QST includes a battery of sensory assessment developed to measure non-painful somatosensation mediated by low-threshold large diameter myelinated afferents (i.e., A-beta fibres) and painful somatosensation small diameter myelinated higher threshold afferents (i.e., A-delta fibres) and small diameter unmyelinated high threshold polymodal afferents (i.e., C-fibres) [12,13,21–24]. The purpose of our study was to evaluate the effect of kinesiology taping on cutaneous somatosensation to noxious and innocuous stimuli administered using QST techniques in pain-free human adults with a non-sensitised nociceptive system. To isolate the effects associated with the elasticity of kinesiology tape, kinesiology taping was compared with taping administered using ‘standard’ (rigid) tape and sham tape controls.

## Methods

### Study design

This was a repeated measures parallel-group randomised controlled laboratory study where QST measurements were taken before and during one of three possible interventions: (i) Kinesiology taping (using RockTape, a proprietary kinesiology tape); (ii) Standard taping (using BSN medical Strappal® tape, a proprietary rigid tape); (iii) Sham taping. This study was approved by the Research Ethics Committee of Leeds Beckett University (reference number 20871).

### Sample size

Sample size was calculated in G*power software v 3.1.9.2 [25] using conservative estimates of small effect size of 0.30, 95% power, 5% Type 1 error, and assumptions of repeated measures within-between interaction ANOVA with six measurements and three groups. The total sample size was estimated to be 48 participants with 16 participants per group. It was decided to recruit 18 participants per group to account for attrition and allow for the possibility of removal of outliers in the dataset for sensitivity analysis.

### Participant enrollment

Healthy participants were recruited by lecture announcements and poster advertisements in Leeds Beckett University. Interested volunteers received a participant information pack including information on eligibility to participate in the study. The exclusion criteria were: (i) under the age of 18 years, (ii) known skin sensitivity (e.g., allergy to adhesive tape), (iii) present history of medical illness including ongoing/undiagnosed pain, (iv) current intake of prescribed or over-the-counter medication, (v) pregnancy, and (vi) unable to comprehend simple instructions in English language. There was no restriction on sex/gender, upper age, ethnicity, nor body mass index, although this was recorded. Volunteers were asked to not consume alcohol or caffeine at least six hours prior to the experiment, and wear clothes that would allow easy access to the skin of the lower back. Prior to signing the informed-consent form, all eligible volunteers were provided with a detailed explanation of the study including the likely risks and advice that they could withdraw consent at any time and without giving a reason. After signing consent, participants provided demographic data (Table 1). Ethnicity was categorised according to the recommended ethnic group survey in England [26]. No incentive or compensation was offered for participating in this experiment.

**Table 1:** Demographic characteristics and anthropometric data of all participants.

Variable		Total	KT Group	ST Group	ShT Group
**Sex (*n*)**	Male	17	6	3	8
	Female	37	12	15	10
**Age (years) (**mean±SD)		23.2±6.4	22.2±5.2	21.0±4.8	26.4±7.8
**BMI (kg/m**^**2**^**) (**mean±SD)		23.6±4.4	23.3±4.1	22.8±4.6	24.9±4.4
**Ethnicity (*n*)**	White British	36	12	16	8
	Other White Background	4	1	1	2
	White & Asian	1	--	1	--
	Indian	4	2	--	2
	Pakistani	1	--	--	1
	African	2	--	--	2
	Arab	5	2	--	3
	Other Ethnic Group	1	1	--	--

Abbreviations: KT, kinesiology taping; ST, standard taping; ShT, sham taping.

### Experimental procedure

**Fig 2:**
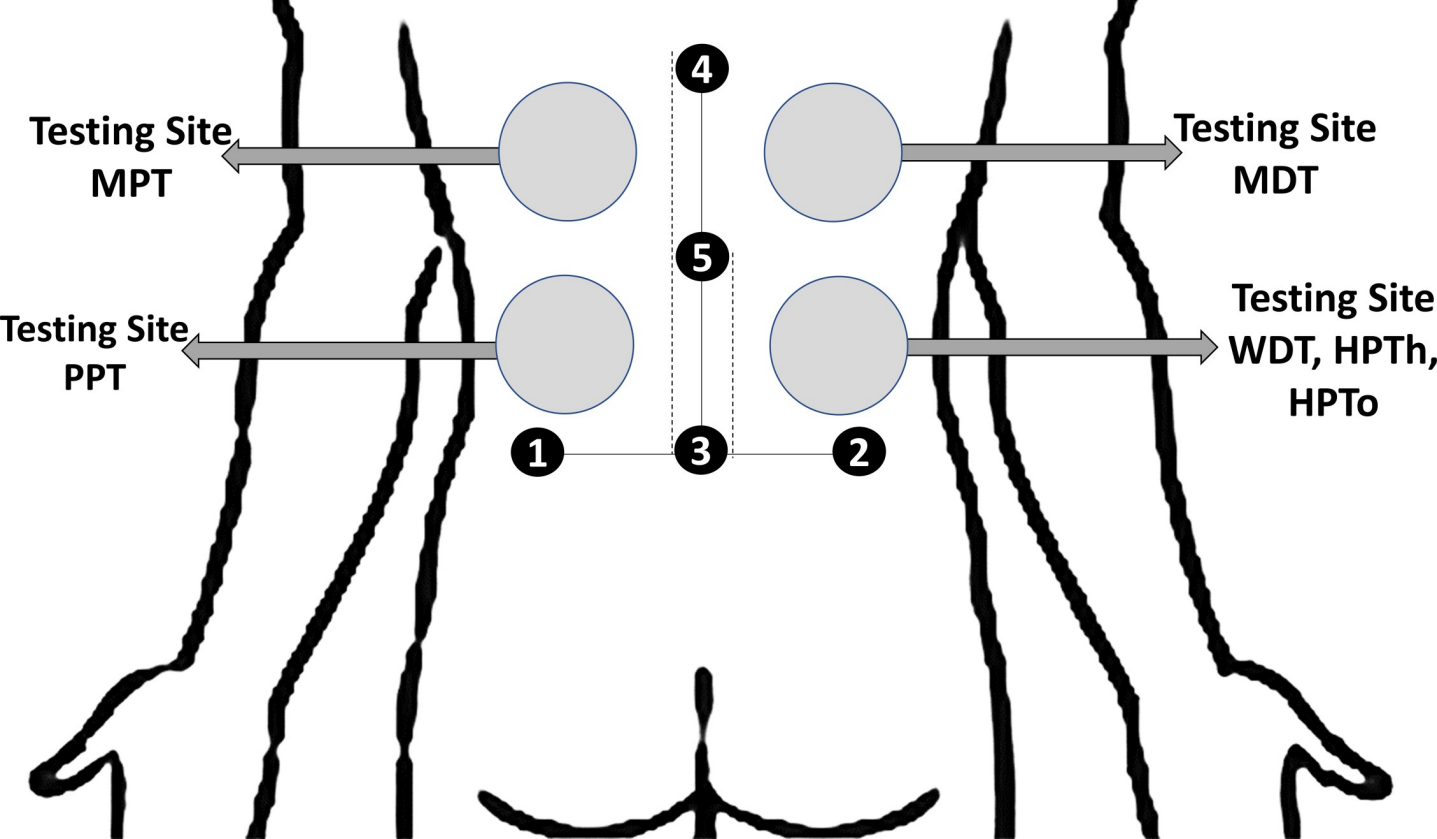
Figure showing sites in the lower back marked for QST measurements and reference points for the application of taping interventions. Points 1 and 2 = superior aspect of left and right iliac crests, respectively; point 3 = L4-5 vertebrae corresponding to the horizontal intercristal line connecting points 1 and 2; point 4 = mid-thoracic spine approximately 30 cm vertically above the point 3; and point 5 midway between points 3 and 4. MPT, mechanical pain threshold; MDT, mechanical detection threshold; PPT, pressure pain threshold; WDT, warm detection threshold; HPTh, heat pain threshold; HPTo, heat pain tolerance.

**Fig 3:**
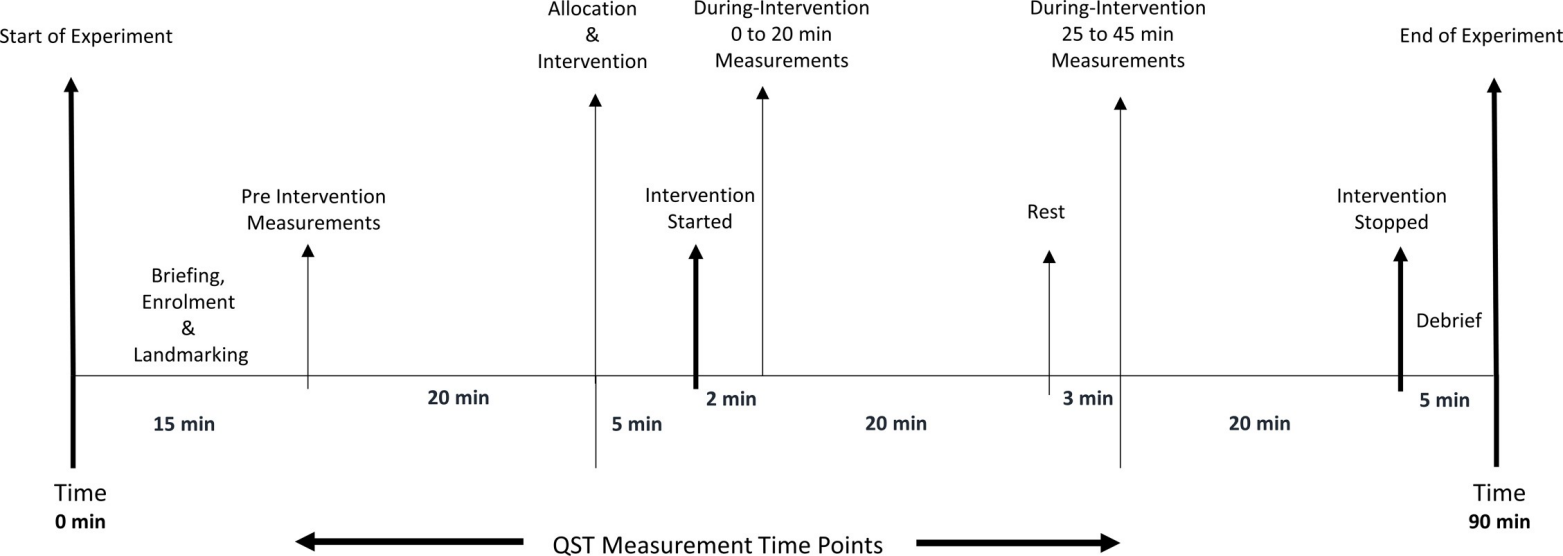
Sequence of events of the experimental procedure.

The experiment, which lasted no longer than 120 minutes, took place in the Pain and Rehabilitation laboratory, Leeds Beckett University (ambient temperature ~23°C) with participants positioned comfortably on a plinth. Participants were familiarised with the experimental procedures [27] with all instructions read verbatim from a crib sheet to ensure that all participants received standardised information. Anatomical landmark points were then marked for the taping interventions as shown in Fig 2. QST measurements were taken before the taping intervention (i.e., pre-intervention) and then twice with the taping intervention applied (i.e., during-intervention with the tape in situ) after which the tape was removed from the skin. The duration of each QST measurement cycle was 20 minutes (Fig 3).

### Randomisation and blinding

Participants were randomised to one of the three intervention groups immediately after the pre-intervention measurement. Constrained block randomisation was used whereby participants selected one of three opaque envelopes that contained a random number generated using a computer software that was associated with a particular intervention. The process of allocation was concealed, however, once the sequence number was revealed, the principal investigator (GB) became aware of the group allocation and subsequently assumed the roles of therapist, data collector and analyst. Interventions were administered to the lower back region so that participants were not able to see taping interventions. This was to promote blinding of the intervention.

### Quantitative sensory testing

The sequence of QST measurements was:

- Warm detection threshold (WDT)
- Heat pain threshold (HPTh)
- Heat pain tolerance (HPTo)
- Mechanical detection threshold (MDT)
- Mechanical pain threshold (MPT)
- Pressure pain threshold (PPT)

Warm detection threshold, heat pain threshold and heat pain tolerance were measured using a thermal sensory analyser (TSA-II, Medoc, Ltd., Ramat-Yishai, Israel) to provide insights to somatosensation associated with A-delta and C fibre function. Mechanical detection threshold was measured using von Frey Filaments (Somedic Aesthesiometer, Sweden) to provide insights to somatosensation associated with cutaneous A-beta fibres and mechanical (sharp) pain threshold measured using a Pinprick Stimulator (MRC, Germany) to provide insights to somatosensation associated with cutaneous A-delta fibres. Pressure pain threshold was measured using a Pressure Algometer (Somedic Algometer, Sweden) to provide insights to somatosensation associated with A-delta and C fibres from cutaneous and deeper-seated tissue [13,21,22,28–30].

### Warm detection threshold, heat pain threshold and heat pain tolerance

A TSA-II using a 30x30 mm thermode probe was applied to the skin corresponding to the right side of thoracic 10/11 vertebrae (T10-T11) dermatomal distribution (Fig 2). Gentle pressure was applied to the probe to ensure its entire surface was in contact with the surface of the skin [22]. The participants sat on a plinth with eyes closed and a pillow on their thighs over which they rested their forearms and leaned forwards. This position allowed a stretch in the soft-tissues of the lower spine.

The sequence of stimuli and measurement protocol was based on Rolke et al. [12]. Three measurements of WDT and HPTh were taken but only one measurement of HPTo was taken to prevent discomfort and the hazard of a thermal burn. The method of ascending limits was used (baseline temperature = 32°C, rate of increase of 1°C/sec, maximum upper limit = 50.5°C, rate of return to baseline = 5^0^ C/sec) with 20 seconds break between measurements. WDT was taken as the point at which the participant felt the slightest change of temperature to warm. HPTh was taken as the point at which the participant felt the first burning or stinging hot sensation (i.e., the first painful sensation due to the rising temperature in the thermode). HPTo was taken as the point at which the participant felt the burning or stinging hot sensation became unbearably painful.

### Mechanical detection, mechanical pain and pressure pain thresholds

Participants lay prone with the head rotated to one side on a plinth with arms extended next to the body or folded near the head. MDT was measured using a standardised set of 17 different von Frey monofilaments (nominal forces of 0.026 to 110 grams). Each monofilament was applied to the skin above the testing site of TSA which corresponded to T8-9 dermatomal distribution (Fig 2). Measurements were taken using the method of limits in sequential ascending and descending orders with approximately 20–30 seconds interval between successive measurements (one ascending and one descending measurement = one testing cycle). The monofilament was applied at a 90^0^ angle to the skin and held for two seconds. The pressure was exerted such that the filaments bowed close to half of its length and the force was recorded at the point where the participant reported a sensation (ascending) or ceased to report a sensation (descending). MDT data in mN units were obtained by measuring the nominal force (in grams) corresponding to the Von Frey filament numbers and then converting the nominal force data to mN. The geometric mean of a series of three consecutive testing cycles was used for data analysis.

Mechanical sharp pain threshold was measured using seven pinprick stimulators with flat contact needle area (diameter 0.25 mm) that exerted forces of 8 to 512 mN. Pinprick stimulators were applied at a 90^0^ angle to the skin for two seconds on the left side corresponding to the dermatomal distribution of T8-9 adjacent to the testing site for measuring MDT (Fig 2). Participants were instructed to report the first ‘sharp’ painful sensation felt as ‘pricking’ or ‘stinging’. Measurements were taken using the method of limits in sequential ascending and descending orders with approximately 20–30 seconds interval between successive measurements (one ascending and one descending measurement = one testing cycle). MPT data in mN units were obtained corresponding to the weights marked on the pinprick stimulators (for example, 32 mN force). The geometric mean of a series of three consecutive testing cycles was used for data analysis.

Mechanical blunt PPT was measured using a pressure algometer (circular probe = 1 cm^2^ diameter) placed on the skin marked on the left side corresponding to the thoracic 10/11 vertebrae (T10-11) dermatomal distribution (adjacent to the testing site of TSA) at a 90^0^ angle and pressure applied (50kPa/s) until participants reported the first sensation of pain (Fig 2). Three measurements were taken with a 20–30 seconds interval between successive measurements. PPT was calculated as the arithmetic mean.

### Taping interventions

Kinesiology tape (RockTape) and standard tape (BSN medical Strappal^®^) were matched for shape (rectangle), length (25 cm), width (5 cm) and colour (plain white). Five strips of both kinesiology and standard tapes were pre-cut into I-shape strips using a measurement tape.

**Fig 4:**
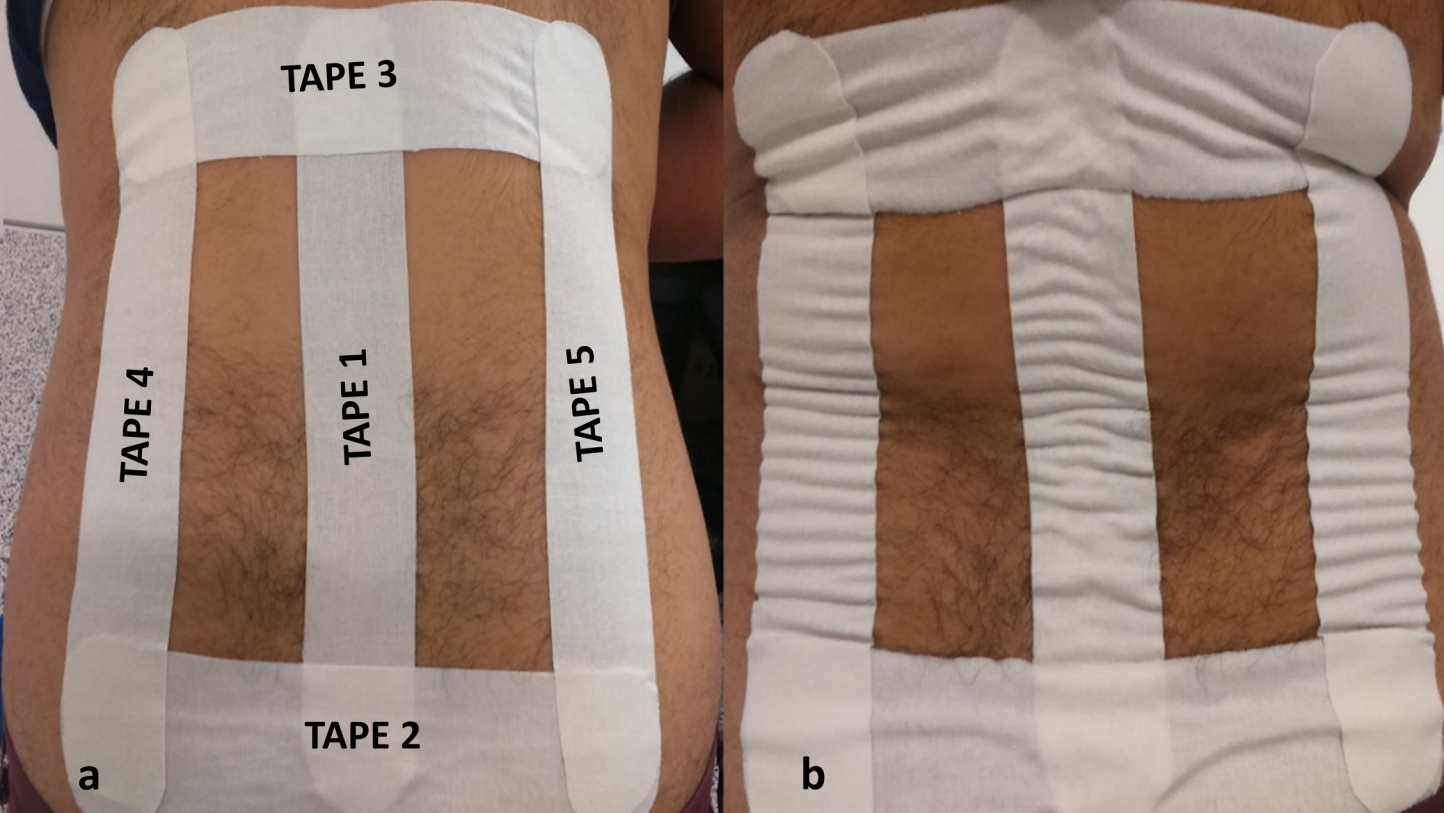
**Image showing application of five strips of kinesiology tape to the skin of the lower back with the participant (a) leaned forwards, and (b) standing straight (with visible convolutions of skin upon kinesiology taping).** Tape 1 was applied vertically long the spine from points 3 (~L4-L5 vertebrae) to 4 as marked in Fig 1. Tape 2 was applied horizontally along the left and right superior iliac crests corresponding to the imaginary intercristal line from points 1 to 2 as marked in Fig 1. Tape 3 was applied horizontally in the direction from right to left overlapping the upper end of Tape 1 in the region of mid-thoracic spine. Tapes 4 and 5 were applied vertically adjoining the upper and lower borders formed by the left and right ends of the two horizontal Tapes 2 and 3, respectively.

Kinesiology tape was applied utilising techniques proposed by Kase et al. (2013) [31] and Rocktape (2017) [32] for reducing / managing pain. Five strips of I-shape kinesiology tapes were applied to the skin of the lower back as shown in Fig 4. The skin and the soft-tissues were brought to a stretch position prior to the application of each strip of kinesiology tape. The two ends of the kinesiology tape (~5 cm) were anchored paper-off tension (i.e., approximately 10–20% inherent stretch after peeling the tape from its paper backing) with the middle segments of the tape applied with an additional stretch of approximately 20%. Three strips of tape (labelled 1, 4 and 5) were applied with the participants in standing and 70 to 90 degrees of lumbar flexion position and the remaining 2 strips of tape (labelled 2 and 3) were applied with the participants in erect standing and left-side spinal rotation position and right-side spinal rotation position, respectively (Fig 4). A measurement tape was used to standardise the length of stretch of kinesiology taping. Procedures recommended by the manufacturers of kinesiology tape for applying the tape were followed. This included cutting/rounding the edges of the tape, applying the ends of the tape off tension and rubbing along the length of tape for approximately 10 seconds to ‘activate’ the heat-sensitive glue for optimal adherence with the skin and removing the tape in the direction of hair growth keeping close to the surface of skin [31,32]. It was not possible to apply kinesiology tapes directly over the QST measurement sites. However, it can be extrapolated from the findings of a study that compared pain-relieving effects of transcutaneous electrical nerve stimulation applied at the same dermatome levels as the site of pain [33] that the application of kinesiology tapes in this study covered the same dermatome levels as the QST measurement areas and would have the potential to trigger modulatory mechanisms of the relevant segment of the nociceptive system.

The standard tape intervention was applied in a similar manner to kinesiology tape (i.e., in erect standing position) except (i) the soft-tissues in the lower back region were not put to stretch prior to taping, and (ii) there was no attempt to stretch the tape. In other words, standard rigid taping was applied without a technique of application unique to kinesiology taping. The standard tape was rubbed to control for the possibility that rubbing kinesiology tape stimulates low-threshold mechanoreceptors.

Participants allocated to the sham taping group did not have any tape applied to their skin. Mock taping procedures were performed in which the investigator pretended to apply two strips of kinesiology tape (25 cm length, 2.5 cm width) horizontally parallel to each other, i.e., one strip of tape across points 1 and 2 and the other strip over the point 4 as shown in Fig 2. However, only the ends (~2.5 cm) of tape’s paper backing was removed and anchored to the skin with the remaining lengths of the tape simply lain over the skin with their protective paper backing not removed. The duration of this procedure was identical to that undertaken for administering kinesiology and standard taping.

### Statistical analysis

Data were entered into Microsoft Excel software (version 2013) to calculate the mean and standard deviation of the demographic characteristics of participants. Data were then imported to SPSS for Windows (IBM Corp, 2013, version 22.0) software for inferential statistical analyses. All the datasets were tested to judge suitability for parametric analysis using mixed ANOVA [34]. Post analyses, except HPTh dataset, all other datasets had to be transformed as certain critical assumptions were violated for mixed ANOVA to produce valid results. WDT and PPT datasets were square root transformed, MDT and MPT datasets were log10 transformed, and HPTo dataset was reflected and then square root transformed. A between-within subjects (mixed) 3 x 3 factorial analysis of variance (ANOVA) were performed on WDT, HPTh, HPTo, MDT, MPT and PPT datasets. The between-subject factors was Intervention (three levels: kinesiology taping, standard taping, sham taping) and the within-subject factor was Time (three levels: pre-intervention, during-intervention-0-20 min, during-intervention-25-45 min). A Greenhouse–Geisser correction was used if Mauchly’s test showed that sphericity could not be assumed. Adjustments were made for multiple comparisons using the Bonferroni correction. Statistical analysis was on an intention-to-treat basis and the significance was set at *p* ≤ 0.05. Partial eta squared (η2_p_) and eta squared (η2) were reported as measures of the estimates of effect size. Where non-significant interactions were found in factorial ANOVA, values of partial eta squared (ratio of the effect variance to the effect plus error variance) were reported along with the main effects of Time and Group. Where significant interactions were found, values of eta squared (ratio of the effect variance to the total variance) derived from one-way ANOVA were reported along with the simple main effects of Time and Group. As a rule of thumb (Cohen), values of partial eta squared of .0099, .0588, and .1379 were used to indicate small, medium and large effects, respectively. Considering that no benchmarks have been suggested to indicate effects corresponding to eta squared values, a comparison of the magnitude of effects between the interventions was made [35].

## Results

### Characteristics of the sample population

Fifty-four participants completed the study (see CONSORT statement, S1 Table). A one-way ANOVA found that there was a significant difference in age (*p =* .028) but not BMI (*p =* .326) across the three groups. Post-hoc test found that the sham taping group was significantly older than the standard taping group (26.4 ± 7.8 years versus 21.0 ± 4.8 years, *p* = 0.028), but there were no statistically significant differences in age between these groups and the kinesiology taping group (22.2 ± 5.2 years, *p* > 0.05). Demographic characteristics and anthropometric data of the participants are presented in Table 1.

### Inferential statistics

Data for mean (*M*) ± standard deviation (*SD*) are presented in Table 2.

**Table 2:** Group x Time data mean and SD counts of WDT, HPTh, HPTo, MDT, MPT and PPT.

		Pre-intervention		During-intervention-0-20-min		During-intervention-25-45-min	
QST Variable	Taping Group	Mean±SD	95% CI (LB/UB)	Mean±SD	95% CI (LB/UB)	Mean±SD	95% CI (LB/UB)
**WDT**	**KT**	35.18±1.12	34.62/35.74	35.00±0.87	34.56/35.43	36.73±1.63	35.92/37.54
	**ST**	34.97±1.10	34.42/35.51	35.07±0.90	34.63/35.52	36.57±1.32	35.91/37.22
	**ShT**	35.04±1.11	34.49/35.59	35.89±1.47	35.16/36.62	36.90±1.80	36.00/37.79
**HPTh**	**KT**	42.57±3.02	41.11/44.03	42.29±3.26	40.85/43.72	44.00±3.28	42.43/45.58
	**ST**	42.28±3.16	40.82/43.75	43.21±2.64	41.77/44.64	44.56±2.95	42.98/46.14
	**ShT**	42.74±3.10	41.27/44.20	43.36±3.16	41.92/44.79	43.40±3.72	41.82/44.98
**HPTo**	**KT**	47.68±2.75	46.31/49.04	47.61±2.78	46.23/48.99	48.27±2.66	46.95/49.59
	**ST**	47.61±2.82	46.21/49.00	48.11±2.18	47.02/49.19	48.64±1.99	47.66/49.63
	**ShT**	47.42±2.16	46.35/48.49	47.71±2.21	46.61/48.80	47.84±2.05	46.83/48.86
**MDT**	**KT**	0.66±0.63	0.35/.98	1.85±1.56	1.07/2.62	3.02±4.28	0.89/5.15
	**ST**	0.81±0.85	0.39/1.23	2.04±1.87	1.11/2.97	2.82±2.67	1.49/4.15
	**ShT**	1.13±0.96	0.65/1.61	1.58±1.71	0.73/2.43	1.90±2.45	0.68/3.12
**MPT**	**KT**	40.33±26.29	27.25/53.41	85.31±59.05	55.95/114.68	98.77±56.09	70.87/126.66
	**ST**	67.20±50.13	42.27/92.13	117.36±93.41	70.91/163.81	142.59±112.84	86.48/198.70
	**ShT**	82.46±64.23	50.52/114.40	97.44±77.52	58.89/135.99	96.33±68.59	62.22/130.44
**PPT**	**KT**	522.24±236.08	415.48/628.99	557.35±226.26	444.83/669.86	589.16±261.00	459.37/718.96
	**ST**	518.26±222.27	407.72/628.79	548.27±254.68	421.62/674.92	557.61±258.08	429.27/685.95
	**ShT**	503.66±201.58	403.41/603.91	522.24±214.68	415.48/628.99	509.35±243.74	388.14/630.56

Abbreviations: QST, quantitative sensory testing; WDT, warm detection threshold; HPTh, heat pain threshold; HPTo, heat pain tolerance; MDT, mechanical detection threshold; MPT, mechanical pain threshold; PPT, pressure pain threshold; KT, kinesiology taping; ST, standard taping; ShT, sham taping; CI, confidence interval; LB/UB, lower/upper bound.

### Warm detection threshold

There was no statistically significant interaction between the intervention and time on WDT (*F*_3.604, 91.910_ = 2.10, *p* = .09, η2_p_ = .076). The main effect of time showed a statistically significant difference in mean WDT at the different time points (*F*_1.802, 91.910_ = 67.78, p < .0005, η2_p_ = .571). Bonferroni post-hoc test results showed that WDT was not statistically significantly different between pre-intervention and during-intervention-0-20 min but was statistically significantly increased at during-intervention-25-45 min compared with pre-intervention, and during-intervention-0-20 min (see S2 Table). The main effect of group showed that there was no statistically significant difference in mean WDT between intervention groups (*F*_2, 51_ = .639, *p* = .532, η2_p_ = .024).

### Heat pain threshold

There was no statistically significant interaction between the intervention and time on HPTh (*F*_3.747, 95.547_ = 2.14, *p* = .09, η2_p_ = .077). The main effect of time showed a statistically significant difference in mean HPTh at the different time points (*F*_1.873, 95.547_ = 11.94, p < .0005, η2_p_ = .190). Bonferroni post-hoc test results showed that HPTh was not statistically significantly different between pre-intervention and during-intervention-0-20 min. HPTh was significantly higher during-intervention-25-45 min than pre-intervention and during-intervention-0-20 min time points (see S2 Table). The main effect of group showed that there was no statistically significant difference in mean HPTh between intervention groups (*F*_2, 51_ = .09, *p* = .92, η2_p_ = .003).

### Heat pain tolerance

There was no statistically significant interaction between the intervention and time on HPTo (*F*_4, 102_ = .83, *p* = .51, η2_p_ = .031). The main effect of time showed a statistically significant difference in mean HPTo at the different time points (*F*_2, 102_ = 7.97, p = .001, η2_p_ = .135). Bonferroni post-hoc test results showed that HPTo was not statistically significantly different between pre-intervention and during-intervention-0-20 min but was statistically significantly lower at during-intervention-25-45 min compared with pre-intervention, and during-intervention-0-20 min (see S2 Table). The main effect of group showed that there was no statistically significant difference in mean HPTo between intervention groups (*F*_2, 51_ = .26, *p* = .77, η2_p_ = .010).

### Mechanical detection threshold

There was a statistically significant interaction between the intervention and time on MDT (*F*_3.480, 88.729_ = 6.19, *p* < .0005, η2_p_ = .195) (see S1 Fig). However, simple main effect for group revealed no statistically significant differences in MDT between interventions at the time point during-intervention-0-20 min (*F*_2, 51_ = .40, *p* = .68, η2 = .015) nor at the time point during-intervention-25-45 min (*F*_2, 51_ = 1.47, *p* = .24, η2 = .055). Tukey HSD post hoc multiple comparisons among treatment groups is provided in S3 Table.

Simple main effect for time revealed statistically significant effect of time on MDT for the kinesiology taping group (*F*_1.402, 23.833_ = 25.21, *p* < .0005, η2 = .597). Bonferroni post-hoc test results showed that MDT was statistically significantly higher at during-intervention-0-20 min compared with pre-intervention, and higher at during-intervention-25-45 min compared with pre-intervention, and during-intervention-0-20 min. There was a statistically significant effect of time on MDT for the standard taping group (*F*_2, 34_ = 29.05, *p* < .0005, η2 = .631). Bonferroni post-hoc test results showed that MDT was not statistically significantly different between during-intervention-0-20 min and during-intervention-25-45 min time points but was statistically significantly increased at during-intervention-0-20 min compared with pre-intervention, and at during-intervention-25-45 min compared with pre-intervention (see S2 Table). There was no statistically significant effect of time on MDT for the sham taping group (*F*_2, 34_ = 2.20, *p* = .13, η2 = .115).

### Mechanical pain threshold

There was a statistically significant interaction between the intervention and time on MPT (*F*_2.960, 75.473_ = 8.28, *p* < .0005, η2_p_ = .245) (see S2 Fig). However, simple main effect for group revealed no statistically significant differences in MPT between interventions at the time point during-intervention-0-20 min (*F*_2, 51_ = .24, *p* = .79, η2 = .009) nor at the time point during-intervention-25-45 min (*F*_2, 51_ = .62, *p* = .54, η2 = .024). Tukey HSD post hoc multiple comparisons among treatment groups is provided in S3 Table.

Simple main effect for time revealed statistically significant effect of time on MPT for the kinesiology taping group (*F*_1.345, 22.868_ = 28.14, *p* < .0005, η2 = .623). Bonferroni post-hoc test results showed that MPT was not statistically significantly different between during-intervention-0-20 min and during-intervention-25-45 min time points but was statistically significantly higher at during-intervention-0-20 min compared with pre-intervention, and at during-intervention-25-45 min compared with pre-intervention. There was a statistically significant effect of time on MPT for the standard taping group (*F*_2, 34_ = 30.59, *p* < .0005, η2 = .643). Bonferroni post-hoc test results showed that MPT was close to but not statistically significantly different between during-intervention-0-20 min and during-intervention-25-45 min time points, but was statistically significantly increased at during-intervention-0-20 min compared with pre-intervention, and at during-intervention-25-45 min compared with pre-intervention (see S2 Table). There was no statistically significant effect of time on MPT for the sham taping group (*F*_2, 34_ = 2.80, *p* = .08, η2 = .141).

### Pressure pain threshold

There was no statistically significant interaction between the intervention and time on PPT (*F*_3.292, 83.939_ = .77, *p* = .52, η2_p_ = .029). The main effect of time showed no statistically significant difference in mean PPT at the different time points (*F*_2, 102_ = 2.37, p = .10, η2_p_ = .044). The main effect of group also showed that there was no statistically significant difference in mean PPT between intervention groups (*F*_2, 51_ = .21, *p* = .81, η2_p_ = .008).

## Discussion

Conventional taping and strapping techniques use rigid tape to provide compression, immobilisation and stabilisation / support to the injured soft tissues and joints keeping in view of the biomechanics in order to alleviate pain and promote recovery [8,36]. Kinesiology taping techniques use elastic adhesive tape to facilitate non-noxious cutaneous afferent input in order to reduce onward transmission of noxious input in the central nervous system (akin to ‘closing the pain gate’). We report the findings of the first study to evaluate the effect of the elastic component of kinesiology taping on somatosensation to non-nociceptive and nociceptive stimuli in otherwise pain-free healthy adult human participants. Our findings suggest that applying tape to the skin alters cutaneous mechanosensation but this is not dependent on the tape elasticity because there were no differences in the increase in MDT and MPT relative to pre-taping baseline between kinesiology and non-elastic taping, although both tapes were superior to sham taping. Our findings also suggest that taping affects perception from stimuli applied to superficial (cutaneous) structures but not deeper tissue because there were no differences in PPT between groups. There were increases in WDT and HPTh (large effect sizes) relative to pre-intervention for all intervention groups. It is likely that the increase in WDT and HPTh was due to physiological habituation resulting from repetitive presentation of non-noxious QST stimuli and/or non-specific effects related to receiving taping treatment (e.g., expectation) rather than the actual tape itself. The finding that HPTo (large effect size) declined over the course of the experiment in all intervention groups may reflect the development of thermal hyperalgesia associated with repeated exposure to noxious thermal stimuli.

The findings suggest that taping influences perceptual response to innocuous stimuli associated with low threshold A-beta cutaneous mechanoreceptor afferents (i.e., MDT) and noxious stimuli associated with higher threshold A-delta mechano-nociceptor afferents (i.e., MPT). The effect sizes of both taping interventions were approximately 125% larger on MDT and MPT when compared with sham taping (see S4 Table). One clinical implication of the decreased sensitivity to low threshold A-beta fibre inputs is that taping may be of therapeutic value in the management of non-sensitised localised mechanical allodynia [37], although it would be prudent to not apply the tape over the site of pain, but around it. Further research would be needed to confirm this.

The findings suggest that the elasticity of the kinesiology tape and thereupon formation of skin convolutions (whilst prone lying) does not influence cutaneous mechanosensation. It was notable that there was no or negligible skin convolution (not visible to the naked eye) upon kinesiology taping when in forward flexion position (Fig 4) whilst measuring cutaneous thermosensation. Whether the absence of visible skin convolutions had any influence on QST thermosensation measurement is not known; nonetheless, the rationale for kinesiology taping in this experiment was to produce traction (caused by the elastic recoil of the tape) on the soft-tissues (to stimulate mechanoreceptors) and not necessarily formation of skin convolutions. Nevertheless, the participants remained at rest throughout the experiment and movement of body parts may be necessary to optimise activation of cutaneous mechanoreceptors. It seems plausible that kinesiology taping stimulates mechanoreceptors during movement of the body when the skin stretches and recoils under the tension of the tape. Some mechanoreceptors are rapidly adapting and only respond at the onset and/or offset of mechanical stimuli (e.g., Pacinian Corpuscles and Peritrichial nerve endings). Other cutaneous mechanoreceptors are slowly adapting and continue to respond for a longer duration of time (e.g., Merkel’s disks and Ruffini's corpuscles) [38]. In this experiment, kinesiology tape was applied with soft-tissues in stretched position in an attempt to maximise stimulation of mechanoreceptors, but further research would be needed to evaluate whether stretch and recoil of the skin mediated by movement whilst kinesiology taping was in situ affects somatosensation.

There are few studies using experimental human pain models that have evaluated the effects of kinesiology taping on somatosensation and pain thresholds in individuals with normally functioning non-sensitised [39] and sensitised [14–20] nociceptive system. Meireles et al. [39] evaluated the effect of kinesiology taping with ~25 to 50% stretch (n = 44) versus kinesiology taping without stretch control (n = 41) on experimentally-induced cold pressor pain threshold, total time of immersion and pain intensity (assessed by visual analogue scale, VAS) and found that kinesiology taping reduced pain regardless of the manner of taping. Studies that evaluated the effects of kinesiology taping on exercise-induced muscle pain and hyperalgesia (DOMS) have produced inconsistent findings [14–20].

Bae et al. [14] compared kinesiology taping (n = 16) with sham taping control (n = 17) and found that kinesiology taping increased heat and cold pain thresholds (assessed by a thermal sensory analyser) and decreased pain (assessed by a VAS) at 24 and 48 hours. Boguszewski et al. [15] compared kinesiology taping (n = 17) with no taping control (n = 17) and found that kinesiology taping reduced pain (assessed by VAS) at 48 hours. Krejci [16] compared kinesiology taping with stockinette sleeve and elastic bandaging controls in a crossover study using 29 participants and found immediate reductions in pain (assessed by numeric pain rating scale, NPRS) following kinesiology taping. Kruszyniewicz et al. [17] compared kinesiology taping (experimental limb) with no taping (control limb) on pain (assessed by VAS) in 20 participants and found that kinesiology taping reduced pain at 5 hours and up to five days.

On the other hand, some investigators found that kinesiology taping did not reduce post-exercise or DOMS-related pain [18–20]. Merino-Marban et al. [18] compared kinesiology taping (experimental limb) with no taping (control limb) on pain (assessed by NPRS) in 28 participants (duathletes) and found that kinesiology taping did not reduce calf pain immediately after the intervention nor after the completion of a duathlon competition. Ozmen et al. [19] compared kinesiology taping with a no taping control in a crossover study using 19 participants and found that kinesiology taping did not reduce pain (assessed by pressure algometry) at 48 hours. Boobphachart et al. [20] compared kinesiology taping (n = 17) with placebo taping (n = 17) and static stretching controls (n = 17) and found that kinesiology taping did not reduce pain (assessed by pressure algometry) at 72 hours.

The findings of the previous studies by Ozmen et al. [19] and Boobphachart et al. [20] in which pressure pain threshold was assessed in participants with DOMS-related pain, together with the findings of the present study in which no effects were found for pressure pain threshold assessed in otherwise pain-free participants, suggest that kinesiology taping does not modulate nociceptive sensations mediated by mechanosensitive afferents in deeper tissues irrespective of the state of sensitisation of the nociceptive system. The finding that the effects of kinesiology taping and standard taping on heat pain was no different to sham taping is interesting as this suggests that the application of tape to the skin did not modulate sensations mediated by thermo-nociceptors and A-delta and C fibre afferents. Thus, stimulation of A-beta cutaneous mechanoreceptors by tape per se did not produce cross-modal modulation. This study was conducted using a sample of healthy human participants in the absence of pathology and / or peripheral or central sensitisation and so the findings are not generalisable to clinical practice in patients with pain, yet the influence of taping on pain with thermal qualities is worthy of further research.

QST is a standardised method to evaluate the function of primary afferent fibres, although limitations regarding the reproducibility and repeatability have been recognised [40]. Measurement errors are likely to have a limited impact on comparisons between groups and are likely to be systematic rather than random as the method for QST was consistent across all participants using a well-defined protocol. One of the criticisms of this study could be the lack of control for extraneous variables including age, sex, ethnicity, and psychosocial status, which can have varying influence on the reporting of pain threshold levels [41]. However, the repeated measures design of the study would account for the differences in demographic characteristics that may cause variability between participants. The absence of assessor blinding and monitoring of participant blinding were also methodological shortcomings. There were no visual clues to aid participants detecting whether or not tape was applied to their skin because tape was applied to the low back. Nevertheless, it seems possible that participants may have been able to determine whether tape was in situ by sensations evoked by the tape is situ. Future research could include post-intervention measurements to determine if the reported values of thresholds during taping intervention return towards baseline, and if the modulation of somatosensation associated with therapeutic tape is specific to the modality of the stimulus, i.e., mechanical rather than thermal.

## Conclusions

In conclusion, the findings of this study provide tentative evidence that applying tape to the skin, irrespective of elasticity in the tape, affects somatosensation in response to innocuous and noxious mechanical stimuli. The findings also suggest that there is no difference in the effects on cutaneous thermal and deep pressure nociception (heat and pressure pain) when comparing kinesiology taping, standard taping and sham taping in response to experimental stimulation of a normal functioning nociceptive system in the absence of sensitisation in otherwise pain-free humans.

## Supporting information

S1 Fig Profile plot showing significant interaction between group and time (MDT). Y-axis: estimated marginal means (transformed data); X-axis: Time.
(TIF)

S2 Fig Profile plot showing significant interaction between group and time (MPT). Y-axis: estimated marginal means (transformed data); X-axis: Time.
(TIF)

S1 Table CONSORT statement. (DOCX)

S2 Table Pairwise comparisons between the three-time points of the analysed data. **For MDT and MPT data set, simple main effects for time are reported.** Bonferroni correction for multiple comparisons. *Mean difference statistically significant at <0.05. Abbreviations: WDT, warm detection threshold; HPTh, heat pain threshold; HPTo, heat pain tolerance; MDT, mechanical detection threshold; MPT, mechanical pain threshold; PPT, pressure pain threshold.
(DOCX)

S3 Table Tukey HSD post hoc test multiple comparisons based on observed means for MDT and MPT dataset (simple main effect for the group). *Mean difference statistically significant at <0.017. Abbreviations: MDT, mechanical detection threshold; MPT, mechanical pain threshold. * the error term is Mean Square (Error) = .13 † the error term is Mean Square (Error) = .19 ‡ the error term is Mean Square (Error) = .22 ** the error term is Mean Square (Error) = .11 †† the error term is Mean Square (Error) = .10 ‡‡ the error term is Mean Square (Error) = .10.
(DOCX)

S4 Table One-way ANOVA (a) Tests of between-subjects effects; (b) Tests of within-subjects effects.
(DOCX)
