# Supplementary figures and images for: The effects of kinesiology taping on experimentally-induced thermal and mechanical pain in otherwise pain-free healthy humans: A randomised controlled repeated-measures laboratory study
Source: PLoS One. 2019 Dec 10;14(12):e0226109. doi: 10.1371/journal.pone.0226109 (PMC6903766; doi:10.1371/journal.pone.0226109)

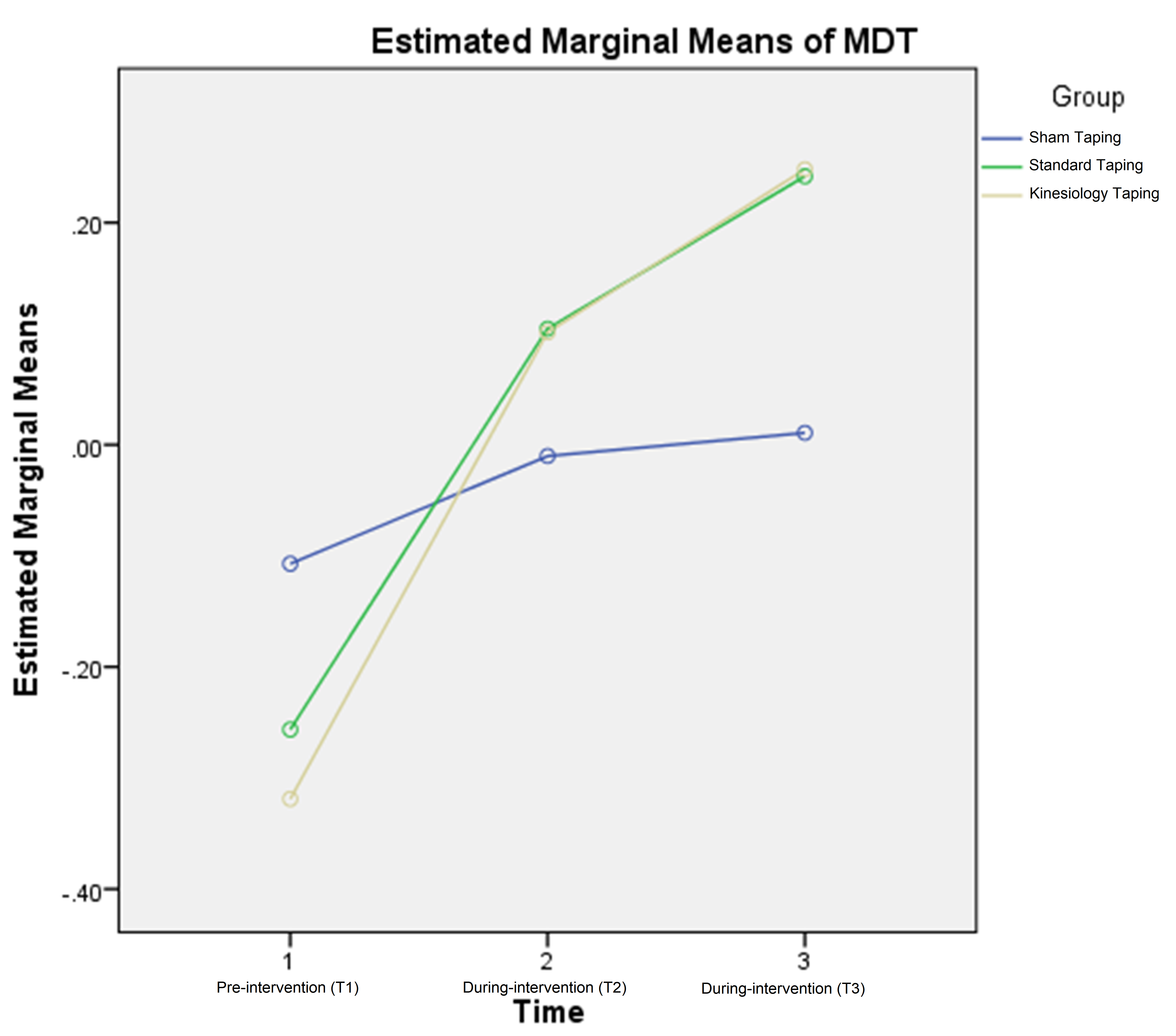

Supplement: S1 Fig — Y-axis: estimated marginal means (transformed data); X-axis: Time. (TIF) [file pone.0226109.s001.tif]

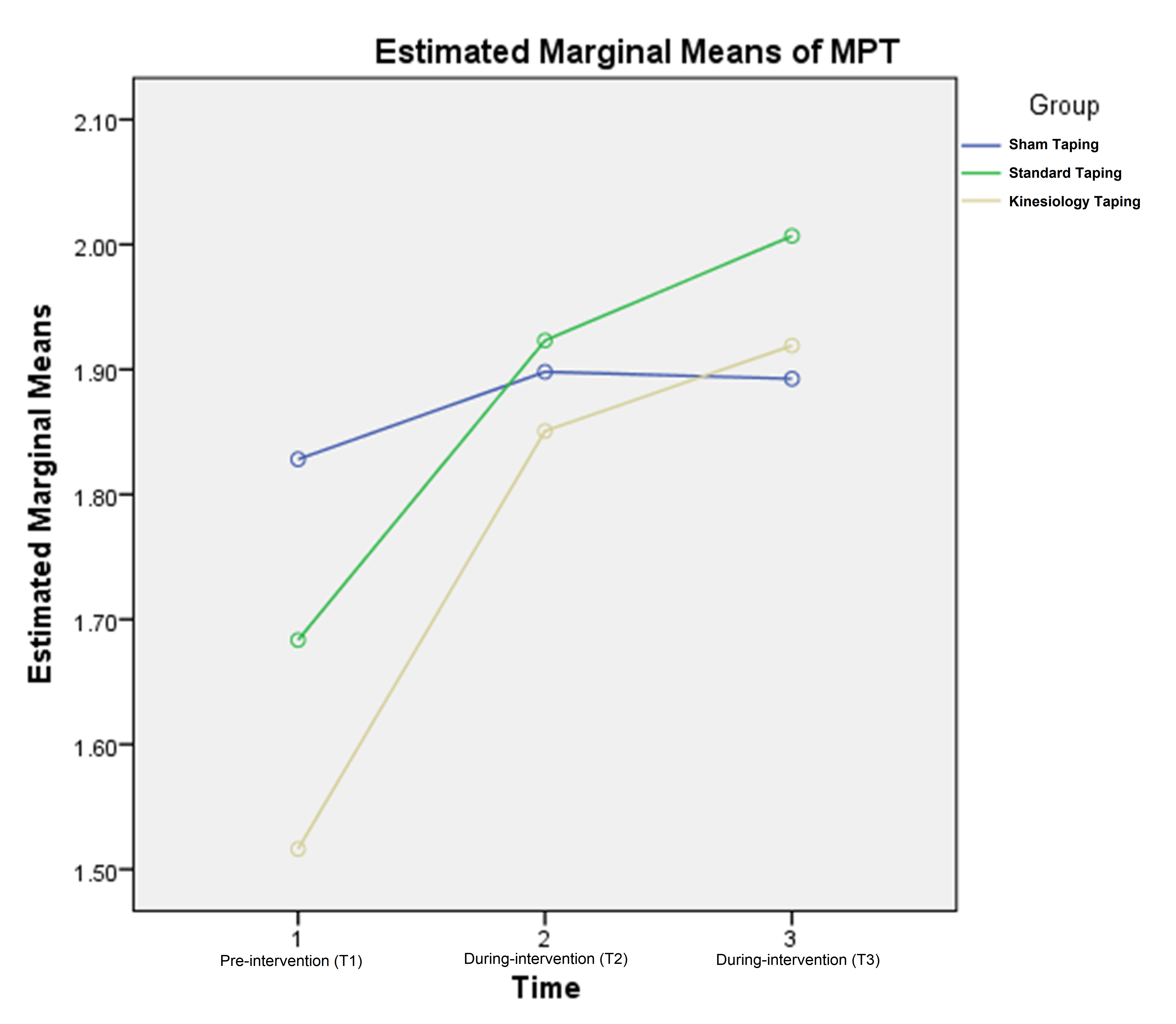

Supplement: S2 Fig — Y-axis: estimated marginal means (transformed data); X-axis: Time. (TIF) [file pone.0226109.s002.tif]
