# Supplementary material for: The effects of kinesiology taping on experimentally-induced thermal and mechanical pain in otherwise pain-free healthy humans: A randomised controlled repeated-measures laboratory study
Source: PLoS One. 2019 Dec 10;14(12):e0226109. doi: 10.1371/journal.pone.0226109 (PMC6903766; doi:10.1371/journal.pone.0226109)
Supplement: S1 Table — (DOCX) [file pone.0226109.s003.docx]

**Enrolment**

Pre-intervention Assessment x 1 (n=54)

**Assessment**

Randomised (n=54)

Assessed for eligibility (n=54)

Excluded (n=0)

Analysed (n=18)
♦ Excluded from analysis (n=0)

Analysed (n=18)
♦ Excluded from analysis (n=0)

Analysed (n=18)
♦ Excluded from analysis (n=0)

**Analysis**

During intervention x 2

During intervention x 2

Allocated to KT (n=18)

♦ Received (n=18)

Allocated to ST (n=18)

♦ Received (n=18)

During intervention x 2

Allocated to NT (n=18)

♦ Received (n=18)

**Allocation**

Table S1. CONSORT statement
