# Supplementary material for: The effects of kinesiology taping on experimentally-induced thermal and mechanical pain in otherwise pain-free healthy humans: A randomised controlled repeated-measures laboratory study
Source: PLoS One. 2019 Dec 10;14(12):e0226109. doi: 10.1371/journal.pone.0226109 (PMC6903766; doi:10.1371/journal.pone.0226109)
Supplement: S2 Table — For MDT and MPT data set, simple main effects for time are reported. Bonferroni correction for multiple comparisons. *Mean difference statistically significant at <0.05. Abbreviations: WDT, warm detection threshold; HPTh, heat pain threshold; HPTo, heat pain tolerance; MDT, mechanical detection threshold; MPT, mechanical pain threshold; PPT, pressure pain threshold. (DOCX) [file pone.0226109.s004.docx]

| **QST**  **Variable** | **Time** | | | **Mean Difference** | **SE** | ***p*** |
| --- | --- | --- | --- | --- | --- | --- |
|  |  |  |  |  |  |  |
| **WDT** | Pre-intervention | | During intervention 0-20 min | -.02 | .01 | .12 |
|  |  |  | During intervention 25-45 min | -.14^*^ | .02 | .00 |
|  | During intervention 0-20 min | | Pre-intervention | .02 | .01 | .12 |
|  |  |  | During intervention 25-45 min | -.12^*^ | .01 | .00 |
|  | During intervention 25-45 min | | Pre-intervention | .14^*^ | .02 | .00 |
|  |  |  | During intervention 0-20 min | .12^*^ | .01 | .00 |
| **HPTh** | Pre-intervention | | During intervention 0-20 min | -.42 | .29 | .45 |
|  |  |  | During intervention 25-45 min | -1.46^*^ | .36 | .00 |
|  | During intervention 0-20 min | | Pre-intervention | .42 | .29 | .45 |
|  |  |  | During intervention 25-45 min | -1.04^*^ | .27 | .00 |
|  | During intervention 25-45 min | | Pre-intervention | 1.46^*^ | .36 | .00 |
|  |  |  | During intervention 0-20 min | 1.04^*^ | .27 | .00 |
| **HPTo** | Pre-intervention | | During intervention 0-20 min | .05 | .05 | .75 |
|  |  |  | During intervention 25-45 min | .17^*^ | .05 | .00 |
|  | During intervention 0-20 min | | Pre-intervention | -.05 | .05 | .75 |
|  |  |  | During intervention 25-45 min | .12^*^ | .04 | .01 |
|  | During intervention 25-45 min | | Pre-intervention | -.17^*^ | .05 | .00 |
|  |  |  | During intervention 0-20 min | -.12^*^ | .04 | .01 |
| **MDT** | Kinesiology taping | Pre-intervention | During intervention 0-20 min | -.42^*^ | .09 | .00 |
|  |  |  | During intervention 25-45 min | -.57^*^ | .10 | .00 |
|  |  | During intervention 0-20 min | Pre-intervention | .42^*^ | .09 | .00 |
|  |  |  | During intervention 25-45 min | -.15^*^ | .05 | .03 |
|  |  | During intervention 25-45 min | Pre-intervention | .57^*^ | .10 | .00 |
|  |  |  | During intervention 0-20 min | .15^*^ | .05 | .03 |
|  | Standard taping | Pre-intervention | During intervention 0-20 min | -.37* | .07 | .00 |
|  |  |  | During intervention 25-45 min | -.50* | .08 | .00 |
|  |  | During intervention 0-20 min | Pre-intervention | .37* | .07 | .00 |
|  |  |  | During intervention 25-45 min | -.14 | .06 | .10 |
|  |  | During intervention 25-45 min | Pre-intervention | .50* | .07 | .00 |
|  |  |  | During intervention 0-20 min | .14 | .06 | .10 |
|  | Sham taping | Pre-intervention | During intervention 0-20 min | -.10 | .05 | .18 |
|  |  |  | During intervention 25-45 min | -.12 | .07 | .39 |
|  |  | During intervention 0-20 min | Pre-intervention | .10 | .05 | .18 |
|  |  |  | During intervention 25-45 min | -.02 | .05 | 1.0 |
|  |  | During intervention 25-45 min | Pre-intervention | .12 | .07 | .39 |
|  |  |  | During intervention 0-20 min | .02 | .05 | 1.0 |
| **MPT** | Kinesiology taping | Pre-intervention | During intervention 0-20 min | -.33* | .06 | .00 |
|  |  |  | During intervention 25-45 min | -.40* | .07 | .00 |
|  |  | During intervention 0-20 min | Pre-intervention | .34* | .06 | .00 |
|  |  |  | During intervention 25-45 min | -.07 | .03 | .16 |
|  |  | During intervention 25-45 min | Pre-intervention | .40* | .07 | .00 |
|  |  |  | During intervention 0-20 min | .07 | .03 | .16 |
|  | Standard taping | Pre-intervention | During intervention 0-20 min | -.24* | .05 | .00 |
|  |  |  | During intervention 25-45 min | -.32* | .05 | .00 |
|  |  | During intervention 0-20 min | Pre-intervention | .24* | .05 | .00 |
|  |  |  | During intervention 25-45 min | -.08 | .03 | .05 |
|  |  | During intervention 25-45 min | Pre-intervention | .32* | .05 | .00 |
|  |  |  | During intervention 0-20 min | .08 | .03 | .05 |
|  | Sham taping | Pre-intervention | During intervention 0-20 min | -.07 | .04 | .18 |
|  |  |  | During intervention 25-45 min | -.06 | .04 | .34 |
|  |  | During intervention 0-20 min | Pre-intervention | .07 | .04 | .18 |
|  |  |  | During intervention 25-45 min | .01 | .02 | 1.0 |
|  |  | During intervention 25-45 min | Pre-intervention | .06 | .04 | .34 |
|  |  |  | During intervention 0-20 min | -.01 | .04 | 1.0 |
| **PPT** | Pre-intervention | | During intervention 0-20 min | -.45 | .28 | .35 |
|  |  |  | During intervention 25-45 min | -.57 | .33 | .28 |
|  | During intervention 0-20 min | | Pre-intervention | .45 | .28 | .35 |
|  |  |  | During intervention 25-45 min | -.12 | .20 | 1.00 |
|  | During intervention 25-45 min | | Pre-intervention | .57 | .33 | .28 |
|  |  |  | During intervention 0-20 min | .12 | .20 | 1.00 |

S2 Table. Pairwise comparisons between the three-time points of the analysed data. For MDT and MPT data set, simple main effects for time are reported.

Bonferroni correction for multiple comparisons.

*mean difference statistically significant at <0.05.

Abbreviations: WDT, warm detection threshold; HPTh, heat pain threshold; HPTo, heat pain tolerance; MDT, mechanical detection threshold; MPT, mechanical pain threshold; PPT, pressure pain threshold.
