# Supplementary material for: The effects of kinesiology taping on experimentally-induced thermal and mechanical pain in otherwise pain-free healthy humans: A randomised controlled repeated-measures laboratory study
Source: PLoS One. 2019 Dec 10;14(12):e0226109. doi: 10.1371/journal.pone.0226109 (PMC6903766; doi:10.1371/journal.pone.0226109)
Supplement: S3 Table — *Mean difference statistically significant at <0.017. Abbreviations: MDT, mechanical detection threshold; MPT, mechanical pain threshold. * the error term is Mean Square (Error) = .13 † the error term is Mean Square (Error) = .19 ‡ the error term is Mean Square (Error) = .22 ** the error term is Mean Square (Error) = .11 †† the error term is Mean Square (Error) = .10 ‡‡ the error term is Mean Square (Error) = .10. (DOCX) [file pone.0226109.s005.docx]

| **QST Measurement** | **Group** | | **Mean Difference** | **SE** | ***p*** |
| --- | --- | --- | --- | --- | --- |
|  |  |  |  |  |  |
| MDT pre-intervention* | Kinesiology taping | Sham taping | -.21 | .12 | .20 |
|  |  | Standard taping | -.06 | .12 | .86 |
|  | Standard taping | Sham taping | -.15 | .12 | .44 |
|  |  | Kinesiology taping | .06 | .12 | .86 |
|  | Sham taping | Standard taping | .15 | .12 | .44 |
|  |  | Kinesiology taping | .21 | .12 | .20 |
| MDT during intervention 0-20 min† | Kinesiology taping | Sham taping | .11 | .15 | .73 |
|  |  | Standard taping | .003 | .15 | 1.00 |
|  | Standard taping | Sham taping | .11 | .15 | .72 |
|  |  | Kinesiology taping | .003 | .15 | 1.00 |
|  | Sham taping | Standard taping | -.11 | .15 | .72 |
|  |  | Kinesiology taping | -.11 | .15 | .73 |
| MDT during intervention 25-45 min‡ | Kinesiology taping | Sham taping | .24 | .16 | .30 |
|  |  | Standard taping | .01 | .16 | 1.00 |
|  | Standard taping | Sham taping | .23 | .16 | .32 |
|  |  | Kinesiology taping | -.01 | .16 | 1.00 |
|  | Sham taping | Standard taping | -.23 | .16 | .32 |
|  |  | Kinesiology taping | -.24 | .16 | .30 |
| MPT pre-intervention** | Kinesiology taping | Sham taping | -.31 | .11 | .02 |
|  |  | Standard taping | -.17 | .11 | .28 |
|  | Standard taping | Sham taping | -.14 | .11 | .38 |
|  |  | Kinesiology taping | .17 | .11 | .28 |
|  | Sham taping | Standard taping | .14 | .11 | .38 |
|  |  | Kinesiology taping | .31 | .11 | .02 |
| MPT during intervention 0-20 min†† | Kinesiology taping | Sham taping | -.05 | .11 | .90 |
|  |  | Standard taping | -.07 | .11 | .78 |
|  | Standard taping | Sham taping | .03 | .11 | .97 |
|  |  | Kinesiology taping | .07 | .11 | .78 |
|  | Sham taping | Standard taping | -.03 | .11 | .97 |
|  |  | Kinesiology taping | .05 | .11 | .90 |
| MPT during intervention 25-45 min‡‡ | Kinesiology taping | Sham taping | .03 | .11 | .97 |
|  |  | Standard taping | -.09 | .11 | .70 |
|  | Standard taping | Sham taping | .11 | .11 | .54 |
|  |  | Kinesiology taping | .09 | .11 | .70 |
|  | Sham taping | Standard taping | -.11 | .11 | .54 |
|  |  | Kinesiology taping | -.03 | .11 | .97 |

S3 Table. Tukey HSD post hoc test multiple comparisons based on observed means for MDT and MPT dataset (simple main effect for the group).

Mean difference statistically significant at <0.017 (adjusted for multiple comparisons).

Abbreviations: MDT, mechanical detection threshold; MPT, mechanical pain threshold.

* the error term is Mean Square (Error) = .13

† the error term is Mean Square (Error) = .19

‡ the error term is Mean Square (Error) = .22

** the error term is Mean Square (Error) = .11

†† the error term is Mean Square (Error) = .10

‡‡ the error term is Mean Square (Error) = .10
