# Supplementary material for: The effects of kinesiology taping on experimentally-induced thermal and mechanical pain in otherwise pain-free healthy humans: A randomised controlled repeated-measures laboratory study
Source: PLoS One. 2019 Dec 10;14(12):e0226109. doi: 10.1371/journal.pone.0226109 (PMC6903766; doi:10.1371/journal.pone.0226109)
Supplement: S4 Table — One-way ANOVA (a) Tests of between-subjects effects; (b) Tests of within-subjects effects. (DOCX) [file pone.0226109.s006.docx]

| **Dependent Variable** | **Time** | **Df, error** | **F** | ***p*** | **Eta Squared** |
| --- | --- | --- | --- | --- | --- |
| MDT | T2 | 2, 51 | .40 | .68 | .015 |
|  | T3 | 2, 51 | 1.47 | .24 | .055 |
| MPT | T2 | 2, 51 | .24 | .79 | .009 |
|  | T3 | 2, 51 | .62 | .54 | .024 |

1. Tests of between-subjects effects

| **Independent Variable** | **Dependent Variable** | **Df, error** | **F** | ***p*** | **Eta Squared** |
| --- | --- | --- | --- | --- | --- |
| Kinesiology taping | MDT | 1.402, 23.833 | 25.21 | .0005 | .597 |
|  | MPT | 1.345, 22.868 | 28.14 | **.**0005 | .623 |
| Standard taping | MDT | 2, 34 | 29.05 | .0005 | .631 |
|  | MPT | 2, 34 | 30.59 | **.**0005 | **.**643 |
| Sham Taping | MDT | 2, 34 | 2.20 | .13 | .115 |
|  | MPT | 2, 34 | 2.80 | **.**08 | **.**141 |

1. Tests of within-subjects effects

S4 Table. One-way ANOVA (a) Tests of between-subjects effects; (b) Tests of within-subjects effects

Abbreviations: MDT, mechanical detection threshold; MPT, mechanical pain threshold; T2, during-intervention-0-20 min; T3, during-intervention-25-45 min
